# Predicting Grade group 2 or higher cancer at prostate biopsy by 4Kscore in blood and uCaP microRNA model in urine

**DOI:** 10.1038/s41598-022-19460-6

**Published:** 2022-09-07

**Authors:** Jacob Fredsøe, Martin Rasmussen, Amy L. Tin, Andrew J. Vickers, Michael Borre, Karina D. Sørensen, Hans Lilja

**Affiliations:** 1 Department of Molecular Medicine, Aarhus University Hospital and Department of Clinical Medicine, Aarhus University, Aarhus, Denmark; 2 Department of Epidemiology and Biostatistics, Memorial Sloan Kettering Cancer Center, New York, NY USA; 3 Department of Urology, Aarhus University Hospital and Department of Clinical Medicine, Aarhus University, Aarhus, Denmark; 4 Departments of Pathology and Laboratory Medicine, Surgery, and Medicine, Memorial Sloan Kettering Cancer Center, 1275 York Avenue, New York, NY 10065 USA; 5 Department of Translational Medicine, Lund University, Malmö, Sweden

**Keywords:** Oncology, Prostate cancer

## Abstract

Elevated prostate-specific antigen (PSA) levels often lead to unnecessary and possibly harmful transrectal ultrasound guided biopsy, e.g. when the biopsy is negative or contains only low-grade insignificant cancer, unlikely to become symptomatic in the man’s normal lifespan. A model based on four-kallikrein markers in blood (commercialized as 4Kscore) predicts risk of Grade group 2 or higher prostate cancer at biopsy, reducing unnecessary biopsies. We assessed whether these results extend to a single institution prostate biopsy cohort of Danish men and are enhanced by three microRNAs from urine (referred to as uCaP). The 4Kscore measured in cryopreserved blood from 234 men referred for 10+ core biopsy to Aarhus University Hospital, 29 with PSA > 25 ng/ml. We explored uCaP in urine from 157 of these men. Combined with age and DRE findings, both 4Kscore and uCaP could accurately predict Grade group 2 or higher prostate cancer (all patients: AUC = 0.802 and 0.797; PSA ≤ 25: AUC = 0.763 and 0.759). There was no additive effect when combining the 4Kscore and uCaP. Limitations include a study cohort with higher risk than commonly reported for biopsy cohorts. Our findings further support the clinical use of the 4Kscore to predict Grade group 2 or higher cancers in men being considered for biopsy.

## Introduction

Prostate cancer is most commonly screened for in men by measurement of prostate-specific antigen (PSA) in blood. While systematic PSA screening clearly reduces prostate cancer mortality, the low specificity of PSA for aggressive disease also leads to overdiagnosis and overtreatment^1–4^. Elevated PSA or a suspicious digital rectal examination (DRE) will commonly prompt transrectal ultrasound (TRUS)-guided biopsies for histopathological evaluation of the presence of any tumors. TRUS biopsy uses a standardized, systematic sampling procedure using 10–12 needles to sample the prostate. However, the suboptimal specificity of PSA for prostate cancer leads to many unnecessary biopsies as well as to detection of many clinically insignificant prostate cancers, which left untreated would not give rise to any symptoms in the man’s normal lifespan^5^. Consequently, a more accurate assessment of the need for performing TRUS biopsy could reduce both unneeded biopsies and reduce overdiagnosis of clinically insignificant cancers.

Previously, a prespecified statistical model based on measurements of four-kallikrein markers (free PSA, intact PSA, total PSA, and kallikrein-related peptidase 2 (hK2)) measured in blood and combined with age and DRE findings, commercially available as the 4Kscore has been demonstrated to accurately predict the presence of Grade group 2 or higher prostate cancer in patients undergoing TRUS biopsy^6–9^. Similarly, a previous study has shown that the expression levels of 3 microRNAs in extracellular vesicle-enriched cell-free urine, when combined in a ratio model (called uCaP) could distinguish between benign prostatic hyperplasia and prostate cancer with greater accuracy than PSA^10^.

In this study, we aimed to investigate (i) the values of the 4Kscore in a single institution cohort of Danish men undergoing initial TRUS biopsy, (ii) whether the uCaP score could predict Grade group 2 or higher prostate cancer (GG2 or above) on biopsy, and (iii) whether a combination of the 4Kscore and uCaP could further enhance predictive accuracy.

## Materials and methods

We identified 240 men (median age 67; quartiles 61–72) who were referred by their general practitioner to Department of Urology, Aarhus University Hospital, Denmark (2015–2018) for initial TRUS guided systematic 10+ core biopsy based on clinical indications (elevated PSA and/or suspect DRE). All research was performed in accordance with relevant guidelines/regulations and in accordance with the Declaration of Helsinki. All patients provided written informed consent and the study was approved by The Central Denmark Region Committees on Health Research Ethics (reference nr. 1-10-72-367-13) and notified to the Danish Data Protection Agency (reference nr. 1-16-02-248-14). Prior to biopsy, the DRE status for each patient was re-evaluated by an urologist and used in the subsequent models. All cores were histopathologically evaluated as part of clinical routine and the highest GG was reported for each patient.

Cryopreserved EDTA plasma samples were shipped on dry ice to Lund University in Malmö, Sweden for measurements of kallikrein levels conducted in 2018–2019 blind to outcome. Total and free PSA levels were measured using the AutoDelfia 1235 automatic immunoassay system using the dual-label DELFIA Prostatus total/free PSA-Assay (Perkin-Elmer, Turku, Finland) calibrated against the World Health Organization (WHO) 96/670 (PSA-WHO) and WHO 68/668 (free PSA-WHO) standards. Intact PSA and human kallikrein-related peptidase 2 (hK2) were measured with F(ab')2 fragments of the monoclonal capture antibodies to reduce the frequency of nonspecific assay interference, as previously reported^11^. We excluded six patients without available kallikrein measurements, leaving us with a final cohort of 234 men.

Our outcome was defined as International Society of Urology Pathologists (ISUP) Grade Group 2 or higher (equivalent to Gleason score 3 + 4 or higher) prostate cancer on biopsy. We compared the kallikrein panel (“4Kscore”) to a “base model” which only included total PSA, age, and DRE results. Coefficients for both models were built on the ProtecT cohort^6^ and were locked down before the data from the current cohort was received, that is, this is an independent validation study of a prespecified model. To evaluate the discriminative accuracy, we calculated the area under the receiver operating curve (AUC) for both the base model and the 4Kscore, and used the Delong, Delong, Clark-Pearson method for inferences on the difference in AUC. To assess the level of agreement between the 4Kscore predictions and the actual risk of Grade group 2 or higher cancer on biopsy we used a calibration plot. Finally, to determine the clinical value of the 4Kscore, we used decision curve analysis to compare the net benefits of this model to the base model, and a biopsy-all and biopsy-none strategy. As the 4Kscore has previously been reported to be useful in men with modestly elevated PSA levels^13^, our primary analysis was carried out in patients with a total PSA value ≤ 25 ng/ml. As a sensitivity analysis, we carried out all the aforementioned analyses after additionally including patients with a total PSA value > 25 ng/ml.

As a secondary aim, we were interested in ascertaining whether the uCaP score could add to the base model to predict Grade group 2 or higher prostate cancer. The uCaP score was calculated based on expression levels of three microRNAs (miR-200b-3p, miR-27b-3p, and miR-30b-5p) measured in first void morning urine collected by patients at their home in a 50-mL falcon tube containing one Stabilur® tablet prior on the day of TRUS biopsy, as described previously^10,14^. We excluded 48 patients without available uCaP score due to either (1) the patient was unable to provide a urine sample (n = 15) or (2) one of the three microRNA assays were below detection limit/failed quality control (n = 33). We created a multivariable logistic regression model with Grade group 2 or higher prostate cancer on biopsy as the outcome, the uCaP score—entered as a non-linear term using restricted cubic splines with knots at the tertiles—as the predictor, and total PSA, age, and DRE as covariates which were included in the model as linear predictors based on coefficients from the ProtecT cohort^6^. As the coefficients for this uCaP model were built on the current dataset, contrasting with the prespecified coefficients for the 4Kscore, which were based on the ProtecT cohort, we utilized repeated tenfold cross validation to evaluate the discriminative accuracy of this model when calculating the AUC. We then used the Delong, Delong, Clark-Pearson method to assess differences in AUC between the uCaP model and the 4Kscore. We additionally included the uCaP model in the decision curve analysis.

Finally, we were interested in determining whether the uCaP score add predictiveness to the 4Kscore. We created a multivariable logistic regression model with Grade group 2 or higher prostate cancer as the outcome, the uCaP score (non-linear) as the predictor and the 4Kscore as a covariate^6^. We then used Wald’s test to assess whether the change in AUC was significant by testing whether the coefficients for non-linear uCaP score variables are simultaneously equal to zero and reported the AUC for this model utilizing repeated tenfold cross validation. All statistical analyses were conducted using STATA 15.0 (StataCorp, College Station, TX).

## Results

We identified 234 patients referred to initial TRUS biopsy with measured total, free and intact PSA as well as hK2 levels in EDTA plasma samples, 29 of whom had a total PSA > 25 ng/ml and were excluded, leaving 205 patients for analysis and whose characteristics are displayed in Table 1. Notably, this cohort is higher risk than is commonly reported for biopsy cohorts^6,15^, with 34% of patients having a positive DRE prior to biopsy and nearly one fifth having extremely high Gleason (GG 4 or 5).

**Table 1:** Patient and clinical characteristics (N = 205). All values are median (quartiles) or frequency (proportion).

	Primary cohort	Patients with available uCaP score
	N = 205	N = 157
Age at blood draw	67 (61, 71)	67 (60, 71)
Total PSA	7.5 (5.6, 11.0)	7.7 (5.8, 11.1)
Free PSA	1.33 (0.90, 1.82)	1.32 (0.90, 1.84)
Intact PSA	0.58 (0.41, 0.80)	0.57 (0.41, 0.81)
hK2-Kallikrein-related peptidase 2	0.07 (0.05, 0.09)	0.07 (0.05, 0.10)
**Biopsy ISUP Grade group**		
No cancer	100 (49%)	74 (47%)
1	28 (14%)	20 (13%)
2	32 (16%)	26 (17%)
3	10 (4.9%)	7 (4.5%)
4	19 (9.3%)	14 (8.9%)
5	16 (7.8%)	16 (10%)
Positive DRE	69 (34%)	53 (34%)

**Figure 1:**
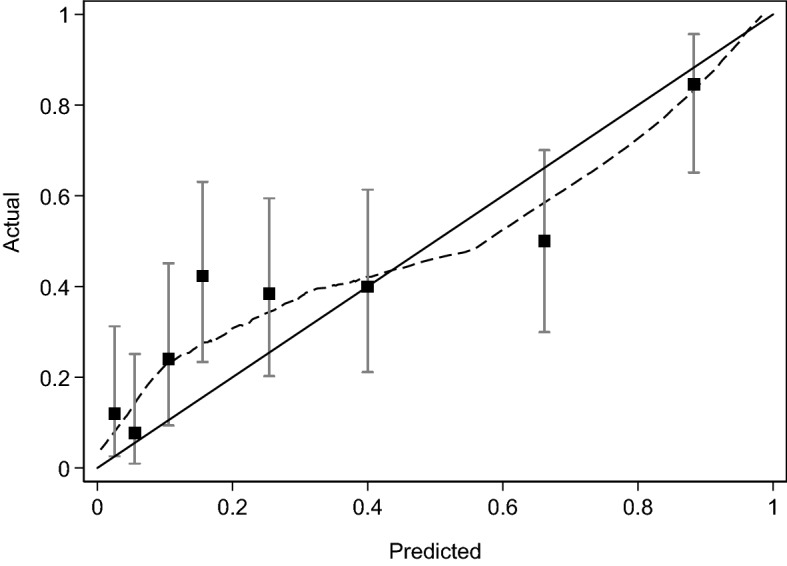
Calibration showing the predicted versus actual Grade group 2 or higher cancer detection using the 4Kscore (Hosmer–Lemeshow goodness-of-fit *p* = 0.085).

**Figure 2:**
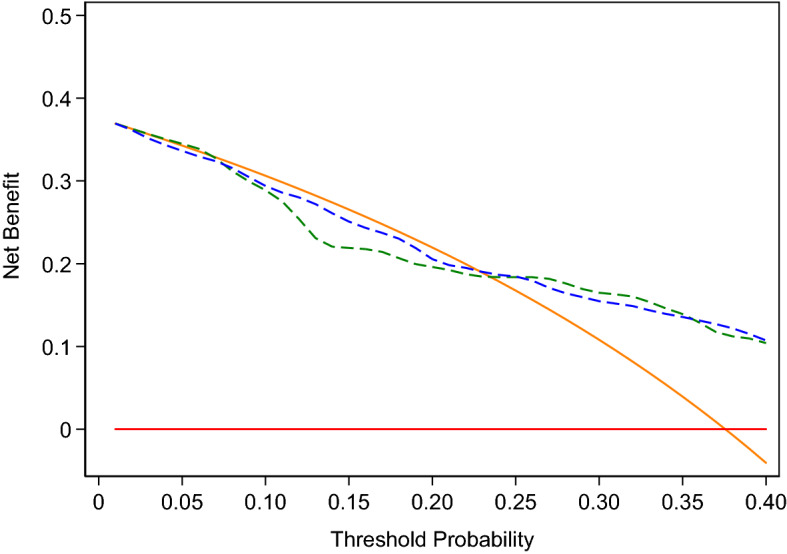
Decision curve analysis comparing the 4Kscore (blue dashed line), base-model (green dashed line), treat-all (orange solid line), and treat-none (red solid line) strategies (N = 205).

On primary analysis, we found that the 4Kscore could predict Grade group 2 or higher prostate cancer in biopsies with high accuracy (AUC 0.763; 95% CI: 0.696, 0.829; Supplementary Table S1). However, this difference in discrimination was not statistically significant compared to the base model consisting of total PSA, age, and DRE (AUC 0.733; 95% CI: 0.661, 0.805; difference 0.030; bootstrapped 95% CI: − 0.020, 0.080; *p*-value = 0.2). Figure 1 depicts the calibration of the 4Kscore, where there appears to be miscalibration, with underestimation of risk at lower probabilities (this is also observed in the base model, Supplementary Fig. S1). However, the Hosmer–Lemeshow goodness-of-fit test *p*-value was 0.085, failing to reject the null hypothesis of good calibration. The decision curve analysis is shown in Fig. 2. As expected from the miscalibration at lower threshold probabilities, both the base model and the 4Kscore are inferior to the strategy of biopsying all men unless the threshold probability of aggressive disease was relatively high (~ 25% or higher).

A sensitivity analysis including patients with high total PSA (n = 29 with PSA > 25 ng/ml) did not importantly affect our results. With a wider range of PSAs in the samples, discrimination for both models were increased but the difference between models was similar, with AUCs 0.802 and 0.780 for the 4Kscore and the base model, respectively (*p*-value = 0.2). Calibration and decision analyses were similar (data not shown). Another sensitivity analysis excluding patients with PSA > 10 ng/ml (n = 61) who therefore were likely to be biopsied independent of 4Kscore, yielded further reduced discrimination, with AUCs 0.723 and 0.695 for the 4Kscore and base model, respectively (*p*-value = 0.5). All results of our sensitivity analyses were consistent with our primary analysis, where we see non-statistically significant higher discrimination in the 4Kscore model compared to the base model, indicating consistent results after excluding patients who are at higher risk and could be argued should undergo TRUS biopsy regardless of their 4Kscore prediction.

**Figure 3:**
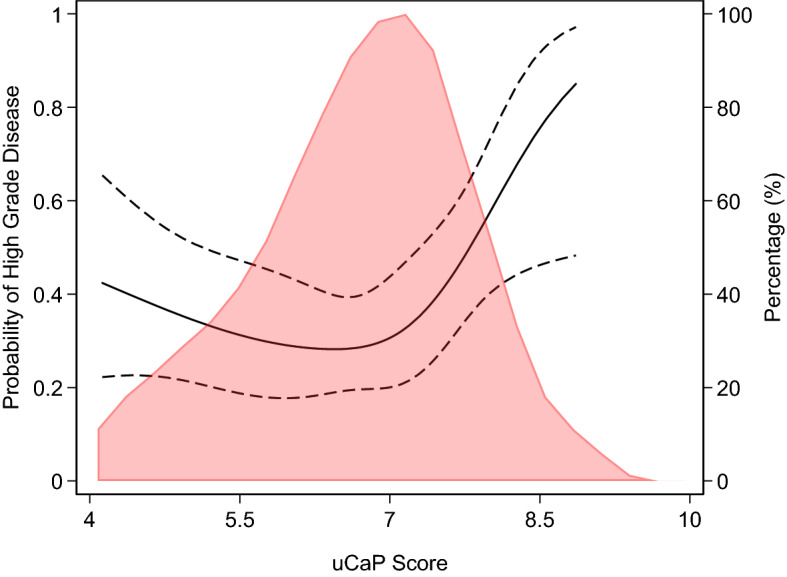
Probability of Grade group 2 or higher disease on biopsy based on uCaP score estimated from the multivariable model with uCaP score, total PSA, age and result of digital rectal exam, (solid line; 95% confidence intervals depicted as dashed lines) overlaid on the distribution of uCaP score.

Among the subset of patients with available uCaP scores (*n* = 157), the median score was 6.8 (quartiles 6.0, 7.4). Patient characteristics among this subset are also shown in Table 1. Figure 3 shows the distribution of the uCaP score, as well as the risk of Grade group 2 or higher prostate cancer on biopsy based on uCaP scores, with covariates set at the mean. We found evidence of an association between the uCaP score and Grade group 2 or higher prostate cancer (non-linear association, overall *p*-value = 0.039) after adjusting for age, DRE, and total PSA. For example, the probability of Grade group 2 or higher prostate cancer is 28% for a patient with an uCaP score of 6.3, and 57% for a patient with the same baseline risk and an uCaP score of 8. The uCaP model had an AUC of 0.759 (95% C.I. 0.680, 0.839), compared to the AUC of 0.758 (95% C.I. 0.682, 0.834) for the 4Kscore (*p*-value > 0.9) in this subcohort of men with available uCaP scores (Supplementary Table S1). Supplementary Fig. S2 shows the decision curve, where the model with the uCaP score has a net benefit equal to or better than other models previously evaluated. After adjusting for the 4Kscore, we did not find evidence of an association between the uCaP score and Grade group 2 or higher prostate cancer and the AUC for this model was 0.766 (95% CI 0.688, 0.844) (non-linear association test *p*-value = 0.092). Supplementary Fig. S3 presents the decision curve, only among men with PSA ≤ 10 ng/ml, as patients with PSA > 10 ng/ml would be biopsied independent of their 4Kscore.

## Discussion

We evaluated the ability of the 4Kscore to detect Grade group 2 or higher prostate cancer in an independent Danish cohort of TRUS biopsy, where we observed high discrimination (AUC = 0.763; 95% CI: 0.696, 0.829). Additionally, when adjusted for age, DRE, and total PSA levels, the urine microRNA model, uCaP had similar accuracy to predict Grade group 2 or higher prostate cancer in TRUS biopsy as the 4Kscore in patients with available uCaP score (AUC = 0.759, 95% CI: 0.680, 0.839; AUC = 0.758 95% CI: 0.682, 0.834; respectively). However, while there was no additional gain in precision by combining the two models (AUC = 0.766, 95% CI: 0.688, 0.844), both models were more accurate than the base model alone (AUC = 0.733; 95% CI: 0.661, 0.805), though this improvement was not statistically significant (test of equality of AUC between base model, 4Kscore, and uCaP model = 0.5).

The ability of the 4Kscore to improve the prediction of Grade group 2 or higher prostate cancer in biopsy naïve men over total PSA alone is in line with previous studies in other cohorts^6,16,17^ in terms of direction. The AUC found here is comparable to a large meta-analysis, which included data from 12 studies and close to 17,000 patients (AUC = 0.81 for detecting GG ≥ 2)^18^. However, the magnitude of the improvement over the base model was smaller as compared with other studies.

The study has potential limitations. The cohort we used here had higher risk than is commonly reported for biopsy cohorts^6,15^, with 34% of patients having a positive DRE prior to biopsy and nearly one fifth having GG 4 or 5. Among the 205 patients, 38% (95% CI: 31%, 45%) had Grade group 2 or higher cancer on biopsy. For comparison, the ProtecT cohort, upon which the coefficient for the 4Kscore was built, reported only 12.9% with Grade group 2 or higher cancer, and < 2% with GG 4 or 5^6^. A possible explanation for this discrepancy could be that in the ProtecT cohort, men were invited for a PSA test (i.e. PSA screening), while the cohort in this study encompassed patients referred by their general practitioner upon clinical suspicion of prostate cancer. This is also reflected in the median PSA levels for patients with positive TRUS biopsy in the two cohorts, with 7.5 ng/ml here and 5.4 ng/ml in the ProtecT cohort.

More recently, multiparametric MRI (mpMRI) with MRI-targeted biopsies has shown improvement for prostate cancer detection over TRUS biopsy^19–21^. This is also reflected in the recently updated European guidelines for prostate cancer^22^, which now recommend mpMRI before biopsy. Consequently, future studies might include patients referred to mpMRI scans, to ensure the 4Kscore and uCaP remains useful in this setting as well. A recent study where the 4Kscore was combined with PiRADS score have suggested that this is indeed the case for the 4Kscore^23^. Nonetheless, a potential criticism of our study may be that we used as an endpoint Grade group 2 or higher prostate cancer on systematic biopsy, whereas many contemporary biopsies are done with MRI-guidance. We have two responses. First, MRI-targeted biopsy is far from universal. Due to lack of equipment and trained personnel, mpMRI is a very limited resource, unavailable at many centers, and still misses some prostate cancers that may be detected by the random TRUS biopsy approach^5,24^. Consequently, TRUS biopsy may be the preferred or only available option at many centers. Second, concerns have been raised that the apparently superior results of MRI may be an artifact of grade inflation^25^. There is evidence that cancers found by MRI-targeted biopsies are not, grade-for-grade, equivalent to those found on systematic. Specifically, high-grade cancers found only on targeted biopsy appear to be far less aggressive^26^. Hence our findings remain robust for the identification of clinically significant cancer. Similar considerations apply to template biopsies.

## Conclusions

In conclusion, our findings provide further support for the clinical use of the 4Kscore to predict Grade group 2 or higher cancers in men being considered for biopsy. Promising results for uCaP warrant confirmatory research.

## Supplementary Information

Supplementary Information.

## Data Availability

The datasets used and/or analyzed during the current study are available from the authors on reasonable request. Data sharing will require additional ethical and data processing agreements under Danish law. We do not have permission to deposit the datasets in a repository. Please contact the corresponding author, Dr Hans Lilja, with any requests.

## References

[1] Hugosson J, Carlsson S, Aus G, Bergdahl S, Khatami A, Lodding P (2010). Mortality results from the Goteborg randomised population-based prostate-cancer screening trial. Lancet Oncol..

[2] Hugosson J, Roobol MJ, Mansson M, Tammela TLJ, Zappa M, Nelen V (2019). A 16-yr follow-up of the European randomized study of screening for prostate cancer. Eur. Urol..

[3] Schroder FH, Hugosson J, Roobol MJ, Tammela TL, Ciatto S, Nelen V (2012). Prostate-cancer mortality at 11 years of follow-up. N. Engl. J. Med..

[4] Schroder FH, Hugosson J, Roobol MJ, Tammela TL, Zappa M, Nelen V (2014). Screening and prostate cancer mortality: Results of the European Randomised Study of Screening for Prostate Cancer (ERSPC) at 13 years of follow-up. Lancet.

[5] Ahdoot M, Wilbur AR, Reese SE, Lebastchi AH, Mehralivand S, Gomella PT (2020). MRI-targeted, systematic, and combined biopsy for prostate cancer diagnosis. N. Engl. J. Med..

[6] Bryant RJ, Sjoberg DD, Vickers AJ, Robinson MC, Kumar R, Marsden L (2015). Predicting high-grade cancer at ten-core prostate biopsy using four kallikrein markers measured in blood in the ProtecT study. J Natl Cancer Inst..

[7] Parekh DJ, Punnen S, Sjoberg DD, Asroff SW, Bailen JL, Cochran JS (2015). A multi-institutional prospective trial in the USA confirms that the 4Kscore accurately identifies men with high-grade prostate cancer. Eur. Urol..

[8] Punnen S, Pavan N, Parekh DJ (2015). Finding the wolf in sheep's clothing: The 4Kscore is a novel blood test that can accurately identify the risk of aggressive prostate cancer. Rev. Urol..

[9] Vickers AJ, Vertosick EA, Sjoberg DD (2018). Value of a statistical model based on four kallikrein markers in blood, commercially available as 4Kscore, in all reasonable prostate biopsy subgroups. Eur. Urol..

[10] Fredsoe J, Rasmussen AKI, Laursen EB, Cai Y, Howard KA, Pedersen BG (2019). Independent validation of a diagnostic noninvasive 3-MicroRNA ratio model (uCaP) for prostate cancer in cell-free urine. Clin Chem..

[11] Vaisanen V, Eriksson S, Ivaska KK, Lilja H, Nurmi M, Pettersson K (2004). Development of sensitive immunoassays for free and total human glandular kallikrein 2. Clin. Chem..

[12] Vaisanen V, Peltola MT, Lilja H, Nurmi M, Pettersson K (2006). Intact free prostate-specific antigen and free and total human glandular kallikrein 2. Elimination of assay interference by enzymatic digestion of antibodies to F(ab')2 fragments. Anal. Chem..

[13] Vickers A, Vertosick EA, Sjoberg DD, Roobol MJ, Hamdy F, Neal D (2017). Properties of the 4-Kallikrein panel outside the diagnostic Gray zone: Meta-analysis of patients with positive digital rectal examination or prostate specific antigen 10 ng/ml and above. J. Urol..

[14] Fredsoe J, Rasmussen AKI, Thomsen AR, Mouritzen P, Hoyer S, Borre M (2018). Diagnostic and prognostic MicroRNA biomarkers for prostate cancer in cell-free urine. Eur. Urol. Focus..

[15] Haese A, Tin AL, Carlsson SV, Sjoberg DD, Pehrke D, Steuber T (2020). A pre-specified model based on four kallikrein markers in blood improves predictions of adverse pathology and biochemical recurrence after radical prostatectomy. Br. J. Cancer.

[16] Vickers AJ, Cronin AM, Aus G, Pihl CG, Becker C, Pettersson K (2008). A panel of kallikrein markers can reduce unnecessary biopsy for prostate cancer: data from the European Randomized Study of Prostate Cancer Screening in Goteborg, Sweden. BMC Med..

[17] Vickers AJ, Cronin AM, Aus G, Pihl CG, Becker C, Pettersson K (2010). Impact of recent screening on predicting the outcome of prostate cancer biopsy in men with elevated prostate-specific antigen: data from the European Randomized Study of Prostate Cancer Screening in Gothenburg, Sweden. Cancer.

[18] Zappala SM, Scardino PT, Okrongly D, Linder V, Dong Y (2017). Clinical performance of the 4Kscore test to predict high-grade prostate cancer at biopsy: A meta-analysis of us and European clinical validation study results. Rev. Urol..

[19] Elkjaer MC, Andersen MH, Hoyer S, Pedersen BG, Borre M (2018). Multi-parametric magnetic resonance imaging monitoring patients in active surveillance for prostate cancer: a prospective cohort study. Scand. J. Urol..

[20] Futterer JJ, Briganti A, De Visschere P, Emberton M, Giannarini G, Kirkham A (2015). Can clinically significant prostate cancer be detected with multiparametric magnetic resonance imaging? A systematic review of the literature. Eur. Urol..

[21] Moldovan PC, Van den Broeck T, Sylvester R, Marconi L, Bellmunt J, van den Bergh RCN (2017). What is the negative predictive value of multiparametric magnetic resonance imaging in excluding prostate cancer at biopsy? A systematic review and meta-analysis from the European Association of Urology prostate cancer guidelines panel. Eur. Urol..

[22] EAU. https://uroweb.org/guideline/prostate-cancer/

[23] Falagario UG, Lantz A, Jambor I, Martini A, Ratnani P, Wagaskar V (2020). Using biomarkers in patients with positive multiparametric magnetic resonance imaging: 4Kscore predicts the presence of cancer outside the index lesion. Int. J. Urol..

[24] Boesen L, Norgaard N, Logager V, Balslev I, Bisbjerg R, Thestrup KC (2018). Assessment of the diagnostic accuracy of biparametric magnetic resonance imaging for prostate cancer in Biopsy-Naive men: The biparametric MRI for detection of prostate cancer (BIDOC) study. JAMA Netw. Open..

[25] Vickers A, Carlsson SV, Cooperberg M (2020). Routine use of magnetic resonance imaging for early detection of prostate cancer is not justified by the clinical trial evidence. Eur. Urol..

[26] Vickers AJ (2021). Effects of magnetic resonance imaging targeting on overdiagnosis and overtreatment of prostate cancer. Eur. Urol..

