# Supplementary material for: Predicting Grade group 2 or higher cancer at prostate biopsy by 4Kscore in blood and uCaP microRNA model in urine
Source: Sci Rep. 2022 Sep 7;12:15193. doi: 10.1038/s41598-022-19460-6 (PMC9452554; doi:10.1038/s41598-022-19460-6)
Supplement: Supplementary file 1 — Supplementary Information. [file 41598_2022_19460_MOESM1_ESM.docx]

Supplementary Material

**Predicting Grade Group 2 or Higher Cancer at Prostate Biopsy by 4Kscore in blood and uCaP microRNA Model in Urine**

Jacob Fredsøe^1^, Martin Rasmussen^1^, Amy L. Tin^2^, Andrew J. Vickers^2^, Michael Borre^3^, Karina D. Sørensen^1,#^ & Hans Lilja^4,#,*^

^1^Department of Molecular Medicine, Aarhus University Hospital and Department of Clinical Medicine, Aarhus University, Aarhus, Denmark.

^2^Department of Epidemiology and Biostatistics, Memorial Sloan Kettering Cancer Center, New York, NY, USA

^3^Department of Urology, Aarhus University Hospital and Department of Clinical Medicine, Aarhus University, Aarhus, Denmark.

^4^Departments of Pathology and Laboratory Medicine, Surgery, and Medicine, Memorial Sloan Kettering Cancer Center, New York, NY, USA; and Department of Translational Medicine, Lund University, Malmö, Sweden

# Co-last authors

*Corresponding author:

Hans Lilja, MD, PhD

Memorial Sloan Kettering Cancer Center

1275 York Avenue

New York, NY 10065

E-mail: liljah@mskcc.org

**Supplementary Table 1.** Discrimination of our various models in their respective cohorts.

| **Cohort (N)** | **Model**  (Included variables) | **AUC** | **95% CI** |
| --- | --- | --- | --- |
| Primary Cohort  (N=205) | Base  (total PSA + age + DRE) | 0.733 | 0.661, 0.805 |
|  | 4Kscore  (4 kallikrein panel + age + DRE) | 0.763 | 0.696, 0.829 |
| Patients  with available uCaP Score (N=157) | Base  (total PSA + age + DRE) | 0.734 | 0.652, 0.816 |
|  | 4Kscore  (4 kallikrein panel + age + DRE) | 0.758 | 0.682, 0.834 |
|  | uCaP  (uCaP score + total PSA + age + DRE) | 0.759 | 0.680, 0.839 |
|  | 4Kscore + uCaP score  (4 kallikrein panel + age + DRE + uCaP score) | 0.766 | 0.688, 0.844 |

**Supplementary Figure 1.** Calibration showing the predicted versus actual Grade group 2 or higher cancer detection using the base ProtecT model (using total PSA, age, and result of digital rectal exam).

**Supplementary Figure 2**. Decision curve analysis comparing the uCaP model (black dashed line), 4Kscore (blue dashed line), base-model (green dashed line), treat-all (orange solid line), and treat-none (red solid line) strategies. (N=157)

**Supplementary Figure 3**. Decision curve analysis comparing the uCaP model (black dashed line), 4Kscore (blue dashed line), base-model (green dashed line), treat-all (orange solid line), and treat-none (red solid line) strategies among men with PSA ≤10 ng/ml. (N=109)
